# Electrochemical Detection of Bisphenol A Based on Gold Nanoparticles/Multi-Walled Carbon Nanotubes: Applications on Glassy Carbon and Screen Printed Electrodes

**DOI:** 10.3390/s24082570

**Published:** 2024-04-17

**Authors:** Maximina Luis-Sunga, Soledad Carinelli, Gonzalo García, José Luis González-Mora, Pedro A. Salazar-Carballo

**Affiliations:** 1 Laboratory of Sensors, Biosensors and Advanced Materials, Faculty of Health Sciences, Universidad de la Laguna, Campus de Ofra s/n, 38071 La Laguna, Spain; mluissun@ull.edu.es (M.L.-S.); jlgonzal@ull.edu.es (J.L.G.-M.); psalazar@ull.edu.es (P.A.S.-C.); 2 Departamento de Química, Instituto Universitario de Materiales y Nanotecnología, Universidad de la Laguna, P.O. Box 456, 38200 La Laguna, Spain; ggarcia@ull.edu.es; 3 Departamento de Ciencias Médicas Básicas and Instituto de Tecnologías Biomédicas, Universidad de La Laguna, 38200 La Laguna, Spain; 4 Instituto Universitario de Neurociencia, Universidad de la Laguna, 38071 Santa Cruz de Tenerife, Spain

**Keywords:** bisphenol A, electrochemical sensors, gold nanoparticles, carbon nanotubes, flow injection analysis

## Abstract

Bisphenol A (BPA) has been classified as an endocrine-disrupting substance that may cause adverse effects on human health and the environment. The development of simple and sensitive electrochemical biosensors is crucial for the rapid and effective quantitative determination of BPA. This work presents a study on electrochemical sensors utilizing gold nanoparticle-modified multi-walled carbon nanotubes (CNT/AuNPs). Glassy carbon electrodes (GCEs) and screen-printed electrodes (SPEs) were conveniently modified and used for BPA detection. AuNPs were electrodeposited onto the CNT-modified electrodes using the galvanostatic method. The electrodes were properly modified and characterized by using Raman spectroscopy, cyclic voltammetry (CV), and electrochemical impedance analysis (EIS). The electrochemical response of the sensors was studied using differential pulse voltammetry (DPV) and constant potential amperometry (CPA) for modified GCE and SPE electrodes, respectively, and the main analytical parameters were studied and optimized. Problems encountered with the use of GCEs, such as sensor degradation and high limit of detection (LOD), were overcome by using modified SPEs and a flow injection device for the measurements. Under this approach, an LOD as low as 5 nM (S/N = 3) was achieved and presented a linear range up to 20 μM. Finally, our investigation addressed interference, reproducibility, and reusability aspects, successfully identifying BPA in both spiked and authentic samples, including commercial and tap waters. These findings underscore the practical applicability of our method for accurate BPA detection in real-world scenarios. Notably, the integration of SPEs and a flow injection device facilitated simplified automation, offering an exceptionally efficient and reliable solution for precise BPA detection in water analysis laboratories.

## 1. Introduction

Bisphenol A (BPA) [2,2-Bis-(4-hydroxyphenyl) propane] is commonly used as an additive in the plastic industry to enhance the material properties of products, including improved durability, anti-impact properties, transparency, lightweight, etc. [1]. Despite these benefits, BPA is classified as an endocrine-disrupting compound that can interfere with the hormone system of the body. The BPA monomer finds widespread use in the agro-food industry for manufacturing polycarbonate plastics, lacquers, and polyacrylate coatings in food cans, etc. [2,3]. Due to its extensive use, there is a risk of BPA exposure through its continuous leaching into foods [4,5] and migration into aquatic environments (sea, rivers, aquifers) from packaging materials or uncontrolled residues [6,7]. Due to the presence of phenols groups and their special arrangement, its structure is analogous to certain hormonal mediators (i.e., diethylstilbestrol and estradiol) and mimics the effects of estrogens, a female sex hormone, resulting in a negative impact on both animals and humans [8,9]. Additionally, it is important to highlight that many phenolics display high toxicity and resilience against abiotic degradation [10]. Ingesting it may lead to hormonal imbalances and disrupt the normal functioning of the endocrine system. Additionally, BPA can affect the production and release of other hormones, such as thyroid hormones, which play a vital role in regulating metabolism and growth. BPA can also interfere with the development and function of reproductive organs, which can have long-term effects on fertility and reproductive health. Among its main effects, BPA may produce alteration in hormone synthesis, and their metabolism and may affect their concentrations in blood. Among the notable effects described in the literature regarding BPA exposure, are a feminization of male fetuses, reduction in testosterone level testicular and epididymal atrophy, early onset of puberty and irregularities in menstrual cycles, and increased probability of developing endometriosis [11,12]. Because of these effects, BPA is widely recognized as an endocrine-disrupting compound (EDC). In addition, other effects in human health reported are obesity, diabetes, cardiovascular and autoimmune diseases, brain disorders, and prostate and breast cancer among others [12,13]. Thanks to the scientific evidence accumulated during the last decades, some countries and international organizations recognized BPA as a toxic and detrimental substance, with Canada (2010) being the first country to regulate its use and implement restrictions on the sale, advertising, and importation of baby bottles containing BPA mixtures. After changes in Canadian regulations, European countries also began to impose restrictions on the use of bisphenol A due to its potential danger. In 2002, the Commission Directive 2002/72/EC authorized the use of bisphenol A for the fabrication of plastic materials and food-contact materials in Europe, which was valid for 9 years [14]. However, a recent regulation, 10/2011/EU, was implemented on 14 January 2011, which set permissible amounts of bisphenol A in food-contact products and articles suitable for human consumption. As a result, a total ban on the fabrication of baby bottles containing BPA was established in the European Union on 1 March 2011, and the importation of such bottles from non-EU countries was prohibited under Directive 2011/8/EU, effective from 1 June 2011.

To measure the concentration of BPA in various samples, several conventional analytical techniques have been employed, such as high-performance liquid chromatography (HPLC) [15], gas chromatography–mass spectrometry (GC-MS) [16,17], fluorescence [18], chemiluminescence [19], and enzyme-linked immunosorbent assay (ELISA) [20]. These methods are accurate and exhibit a high level of sensitivity, but they can be high priced, unsuitable for on-site analysis, time-consuming, and require specialized equipment. Recently, electrochemical sensors have arisen as a valuable alternative for BPA detection owing to their high sensitivity, low cost, and portability. Therefore, amperometric and voltammetric methods under different approaches such as differential pulse voltammetry (DPV), cyclic voltammetry (CV), linear sweep voltammetry (LSV), and square wave voltammetry (SWV) have been widely used for detecting BPA and other contaminants such as heavy metals [21,22]. Moreover, within the realm of electrochemical methodologies, DPV holds significant prominence. Specifically, DPV has proven effective in identifying heavy metals, BPA, phenolic derivates, paracetamol, and dopamine, among others, with very low limits of detection [23,24].

**Scheme 1:**
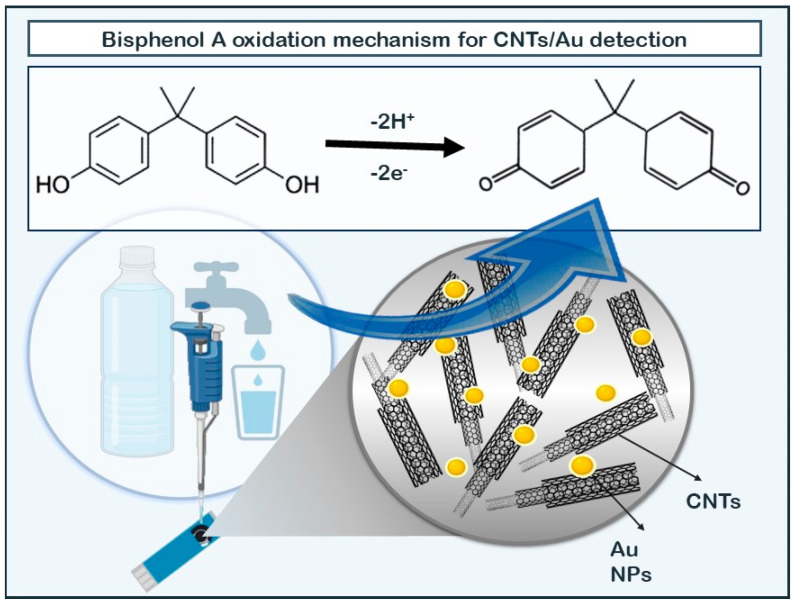
Oxidation mechanism proposed for bisphenol A (BPA).

The determination of BPA through electrochemistry is based on the electroactive nature of the phenolic groups within the molecule. BPA undergoes electrooxidation through a two-electron and two-proton mechanism (see Scheme 1), initiated by the oxidation of the phenolic groups within its structure [3,25,26]. Unfortunately, the oxidation products of BPA cause electrode surface fouling and obstruct the active surface area of electrodes, slowing down the electrode reactions and limiting the reusability of the electrodes. Such difficulties hinder the implementation of this approach in routine analyses. To address this problem, electrode surface modification (with organic, organic–metal electroactive, and inorganic substances, polymer films, metals and metal oxide nanoparticles, and nanocomposites) has been extensively utilized to prevent or reduce electrode surface fouling. Furthermore, different methods and protocols have been developed for regenerating the electrode surface after BPA detection using different organic solvents. However, the last ones can be tedious and often result in low reproducibility.

The detection of phenolic derivatives often relies on various analytical techniques [27]. While techniques like HPLC and spectrophotometry are common, they pose challenges such as complexity, time consumption, and the need for expensive equipment and skilled personnel. Electroanalytical methods are preferred due to their simplicity, user-friendliness, and enhanced sensitivity, making them cost-effective alternatives [28]. Electroanalytical methods offer superior performance, being straightforward, sensitive, and economical. Nonetheless, bare electrodes may not always be ideal due to issues like high overpotential and fouling [29]. Incorporating redox-active materials onto the electrode surface can mitigate these challenges.

Carbon nanotubes (CNTs) and gold nanoparticles (AuNPs) are widely utilized in biosensing applications. The synergistic effect between CNTs and AuNPs on electrodes can be analyzed by considering several key aspects of the materials and their interaction at the electrode–electrolyte interface. Firstly, CNTs are renowned for their high electrical conductivity and large surface area, while AuNPs exhibit high catalytic activity, resulting in a unique and recognized platform for electrochemical sensing [30]. Additionally, the porous structure of CNTs provides excellent electron loading and unloading capability, contributing to a fast and accurate electrode response to changes in analyte concentration. This property can be critical for applications requiring real-time detection and continuous monitoring of chemical species in complex and dynamic environments [31]. Moreover, the presence of AuNPs on the surface of CNTs can enhance the adsorption and catalytic activity of analytes, resulting in increased sensor sensitivity and selectivity [32]. Another critical aspect to consider is the chemical and mechanical stability of the proposed electrode. CNTs and AuNPs act complementarily, improving resistance to corrosion and material degradation, and ensuring consistent and reliable performance over time [33]. This stability is essential for practical applications where prolonged operation and minimal sensor degradation are necessary. For instance, varying the ratio between CNTs and AuNPs, as well as the size and shape of the gold nanoparticles, can be optimized to maximize sensor sensitivity and stability under different operating conditions [30,32].

Furthermore, the integration of CNTs and gold nanoparticles with screen-printed electrodes (SPEs) presents a highly promising avenue for biosensing, owing to its practicality and adaptability. SPEs offer distinct advantages, including their cost-effectiveness derived from mass manufacturability and disposability, as well as their versatility in selectivity and minimal sample requirements across a diverse range of analytes and samples [31]. This integration with flow injection analysis (FIA) represents a notable advancement in enhancing the analytical performance of techniques employing novel electrochemical sensors. The combination of disposable SPEs with CNTs and Au, together with the implementation of an FIA, offers a promising option for biosensing. This option stands out for its operational simplicity, reduced cost of analysis, high sampling frequency, adequate precision, and the ability to easily study the kinetics of chemical reactions.

**Scheme 2:**
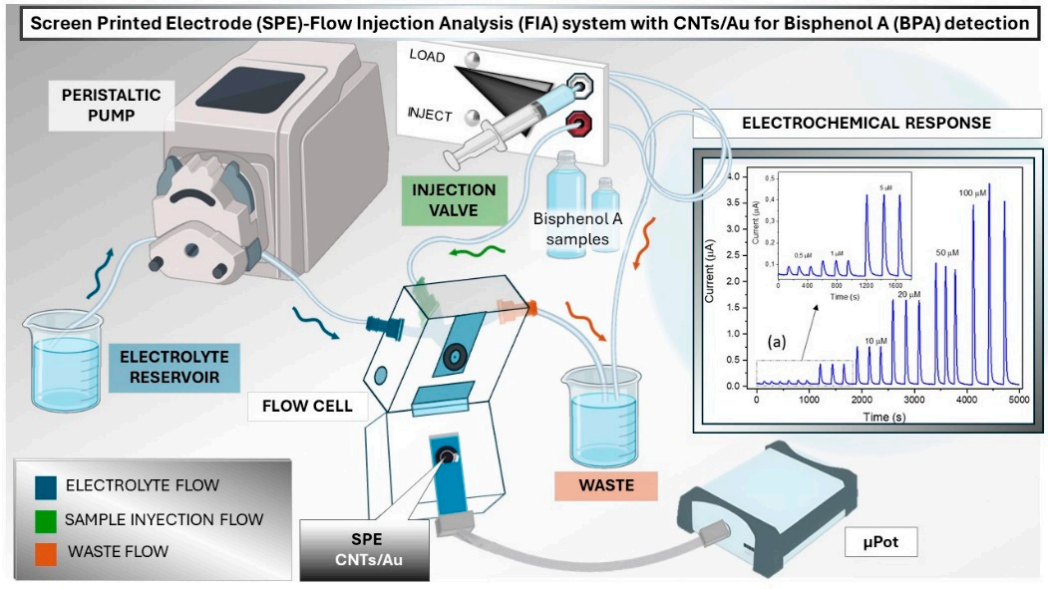
Flow injection analysis coupled with screen-printed electrodes for BPA detection using CNTs/Au.

The FIA coupled with SPEs is an automated system in which a sample is injected into a continuous flow of a carrier (usually a liquid), which transports the sample through the SPE’s sensing area. Additional strategies can be implemented in these systems to minimize problems, such as applying rapid electrochemical cleanups or reactivation of the electrode surface [34]. Scheme 2 illustrates the sensor system based on CNTs/Au on SPE using an FIA system. The process starts with the injection of the sample into the continuous flow of the liquid carrier by means of a pump and an injection valve. The sample, upon injection, binds to the moving electrolyte and is transported through the system. As the mixture of sample and electrolyte flows, it encounters the SPE. At this stage, specific electrochemical reactions are triggered between the sample and the SPE generating an electrochemical signal proportional to its concentration in the sample. The potentiostat captures and quantifies the electrochemical signal generated at the SPE electrodes, translating it into interpretable data for quantitative analysis [35].

This combination of flow processes with nanomaterials not only opens new perspectives in analytical and synthetic fields but also drives progress across various scientific and technological domains. This synergy contributes to both efficiency and sustainability in water analysis while fostering the development of innovative approaches in chemical research and biosensor design.

Herein, we present a novel electrochemical sensor based on a gold-modified carbon nanotube nanocomposite. Various electrodes (glassy carbon (GCE) and screen-printed (SPE) electrodes), nanomaterial configurations (ranging from the simplest to hybrid configurations), and different amperometric methods were employed. The main drawbacks, optimizations, and results are discussed. To overcome the main difficulties encountered in the initial stage of the research (low sensitivity, electrode passivation, low response, fouling effects, time-consuming regeneration, and low reproducibility) with conventional GCE, modified SPEs were used and coupled with a flow injection analysis (FIA) system. This approach allowed for the automated and high-throughput analysis of BPA with high reproducibility and sensitivity, and very low LOD. The reusability of the sensor over several hours and its introduction in an FIA system allows the routine determination of BPA. Additionally, interference tests, recovery tests, and the BPA content of some commercial water and tap water samples are shown. All these results confirm the ability of such an approach for the automated and rapid determination of BPA in large numbers of samples in routine control analysis.

## 2. Materials and Methods

### 2.1. Reagents

Tetrachloroauric (III) acid trihydrate (HAuCl_4_·3H_2_O), BPA, HCl, NaOH, Na_2_HPO_4_, NaH_2_PO_4_, and all interference compounds were purchased from Sigma-Aldrich. Carboxylated multi-walled carbon nanotubes were acquired from Orion High Technologies S.L. Screen-printed carbon (DRP-110) (φ~0.13 cm^2^) and glassy carbon electrodes (φ~0.07 cm^2^) were purchased from DropSens and CH Instruments Inc., respectively. SPCE consisted of a three-electrode system, in which working (4 mm diameter) and counter electrodes are made of carbon and the pseudo-reference electrode is Ag/AgCl. For experiments with GCEs, a platinum wire was used as the counter electrode, meanwhile, an Ag/AgCl (3M KCl) electrode was used as the reference electrode.

Commercial water samples were acquired from local markets and tap water from local suppliers. Carboxylated CNTs (10 mg) were dispersed in 10 ml of water and subjected to sonication for one hour. Before using, CNT solution was sonicated for 1 min. Then, gold nanoparticle precursor solution was obtained dissolving HAuCl_4_·3H_2_O in double-distilled water at a concentration of 3 mM. Both solutions were conserved in the fridge at 4 °C before use. 

### 2.2. Sensor Assembly

GCEs were prepared by polishing with alumina slurry and then washed with sonicated water and acetone. Subsequently, the cleaned GCEs were dried using a stream of nitrogen (N_2_). SPEs were used as received without previous treatment. CNTs were deposited using the drop-casting method, different volumes of the CNTs solution were used (2–10 µL) and the sensor response was optimized. Modified electrodes were dried at room temperature overnight. For gold electrodeposition, a galvanostatic method was used with a current of −25 µA. The deposition time (30–180 s) was optimized for improving the sensor response. Modified SPEs were modified using the same protocol and were assembled for FIA experiments.

### 2.3. Electrode Measurements and Nanocomposite Characterization

The electrochemical measurements, including CV and CPA, as well as electrochemical impedance spectroscopy (EIS), were performed using a PalmSens4 potentiostat and PSTrace5 software Version 5.5.2030 Biuld 18456 t (PalmSens, Houten, The Netherlands). The entire series of electrochemical experiments was carried out using a conventional three-electrode configuration using a Pt electrode (counter electrode), Ag/AgCl (3M) electrode as the reference electrode, and GCE as the working electrode. For SPEs, the complete electrochemical cell contained in the SPE was used. Optimization tests and the analytical measurements were executed in 0.1 M phosphate buffer solution (PBS) at pH 7. The study of the influence of pH was performed in 0.1 M PBS in a pH range of 4–9. Moreover, the impedance analyses were conducted using a ferro/ferricyanide redox couple with a concentration of 0.1 M KCl. The stabilization potential was maintained at the same value of +0.2 V against the reference electrode, and the signal amplitude was set to 10 mV in a frequency range of 0.1–10^5^ Hz. For FIA experiments a methacrylate (ref. FLWCL) low dead volume wall-jet flow cell and peristaltic pump adjustable (0.005 mL/min–30 mL/min) were used, both devices were supplied by Dropsens. The flow rate was optimized to optimize the sensitivity, reproducibility, and time for results. Raman measurements were carried out using an SPELEC RAMAN (Metrohm DropSens) instrument equipped with a green laser source (λ = 532 nm). The system was controlled using DropView Spelec 3.2.2 software. Lastly, scanning electron microscopy (SEM) and energy-dispersive X-ray spectroscopy (EDX) were obtained using a ZEISS EVO 15 microscope.

## 3. Results and Discussion

At first, GCEs were modified with CNTs and gold NPs following the procedure described above. The key parameters involved in the construction were sequentially revised such as CNT amount, and deposition time for gold electrodeposition. To this end, the sensitivity of BPA was assessed for different configurations. After checking, these parameters were fixed as CNTs volume for casting: 6 µL, time for electrodeposition: 60 s. Figure S1 (in the Supplementary Material) shows the typical potential transient obtained during galvanostatic metallic deposition which involves nucleation, growth, and diffusion-limited growth [36,37,38]. In the first region, the potential drops quickly due to the creation of small gold nuclei on the electrode surface, and after that the potential remains relatively constant as the electric current is used to grow the gold deposit. Finally, in the diffusion-limited growth region, the potential starts to slowly increase as the concentration of gold ions decreases on the electrode surface (this effect becomes more evident with longer electrodeposition times).

### 3.1. Raman, Scanning Electron Microscopy, and Energy-Dispersive X-ray Characterization of the CNT/Au Composite

**Figure 1:**
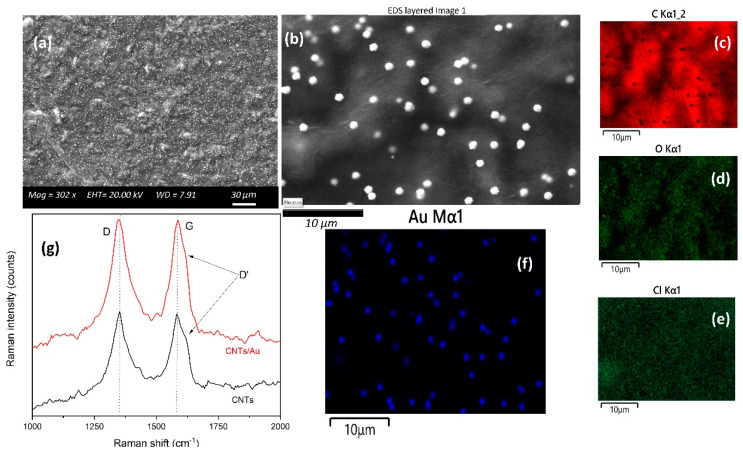
(**a**) SEM image for CNTs/Au hybrid nanocomposite. (**b**) Region of interest (ROI) used for energy-dispersive X-ray (EDX) mapping. Mapping for different elements in the ROI: carbon (**c**), oxygen (**d**), chlorine (**e**), and gold (**f**)**.** (**g**) Raman spectra of pristine CNTs, and CNTs/Au hybrid nanocomposite.

The chemical components of the CNT/Au nanocomposite were corroborated by EDX microanalysis/mapping obtained through SEM analysis (Figure 1a). It provides further confirmation regarding the characteristics of the composite under study. Figure 1b depicts the region of interest under examination. Specifically, Figure 1c corresponds to carbon signals from carbon nanotubes, Figure 1d to oxygen, Figure 1e to chlorine, and Figure 1f to gold nanoparticles in the compositional analysis. The obtained results from the elemental analysis revealed the chemical composition of the sample, indicating that approximately 96% of the composition is carbon (CNTs) and 4% is gold (AuNPs). Such a result was confirmed by XRD analysis. Figure S2 shows the characteristic (111) diffraction peak for the (111) plane of gold.

In addition, Raman spectroscopy was utilized to study the interaction of gold electrodeposition on CNTs. Figure 1g depicts Raman spectra for pristine CNTs (black line) and gold-modified CNTs. In this figure, three contributions can be clearly discerned. The peaks at ca. 1360 cm^−1^ (D band) are correlated to sp^3^ carbon domains, 1580 cm^−1^ (G band) is associated with sp^2^ bonds into the graphitic lattice, and the D band has the corresponding overtone at ca. 3000 cm^−1^ [38]. Interestingly, gold electrodeposition only produces an increment of the carbon signals (known as SERS effect: *surface-enhanced Raman spectroscopy*) and the intensity ratio of G and D bands remains equal. 

### 3.2. Characterization and Analytical Properties of GCE/CNT/Au Electrodes

**Figure 2:**
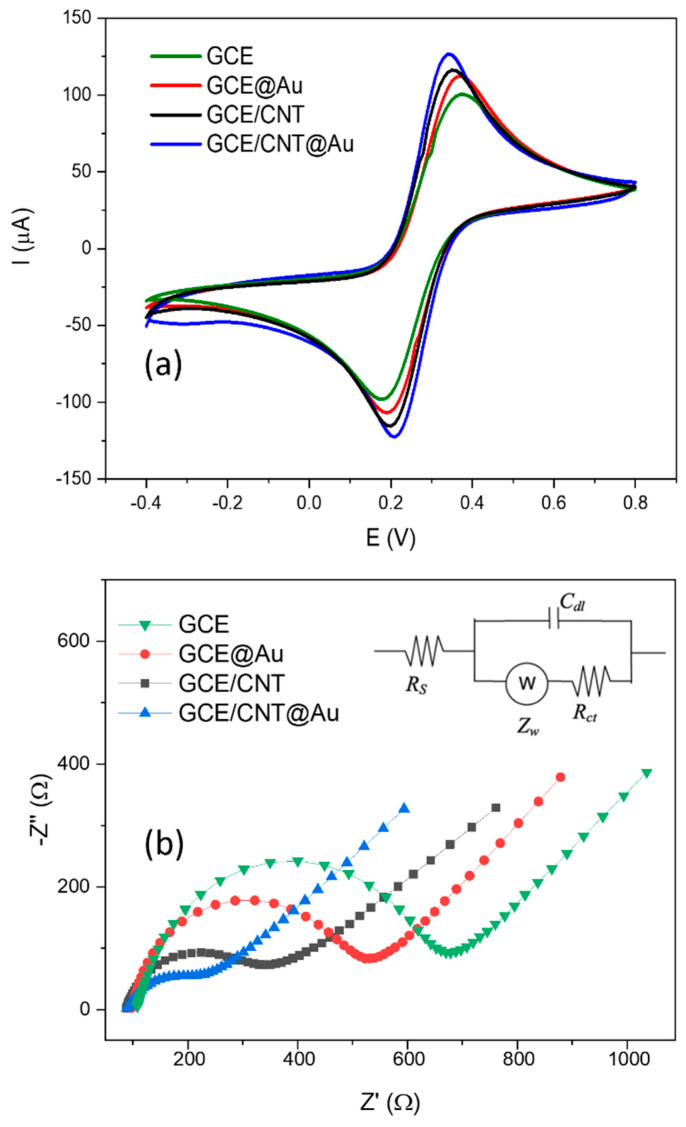
Cyclic voltammograms (**a**) and Nyquist plots (**b**) of bare GCE, GCE/Au, GCE/CNT, and GCE/CNT/Au electrodes in 0.1 M KCl with 5.0 mM [Fe(CN)_6_]^3−/4−^. *Inset: equivalent circuit used for EIS fitting*.

The electrocatalytic properties of the different sensors and their sensitivity against BPA were revised from the basic (GCE) to the final configuration (GCE/CNT/Au), including other configurations: GCE/CNT and GCE/Au. Figure 2 shows the voltammograms and the electrochemical impedance spectroscopy (EIS) results obtained in a 5 mM ferro/ferricyanide solution. In Figure 2a, all electrodes exhibit the characteristic pair of peaks of Fe^2+/3+^ centered ca. 0.22 V. The GCE shows the lowest peak current and the highest peak separation, confirming the worst electrocatalytic properties. However, the integration of CNTs and Au NPs in the design of the sensor improves their electrocatalytic properties. The best results were obtained for the hybrid configuration (GCE/CNTs/Au) confirming the beneficial effect of combining such nanomaterials with high conductivity and surface-to-volume ratio in the BPA sensor design. EIS was further used to investigate the electrochemical properties of the sensor interface such as the charge transfer resistance and double-layer capacitance. Figure 2b shows the Nyquist plot for the GCE/CNT/Au electrode (the results for GCE, GCE/Au, and GCE/CNT electrodes are also illustrated). The impedance spectra of electrochemical sensors typically exhibit two distinct regions: a linear line portion at lower frequencies, which is associated with the diffusion phenomenon, and a semicircular portion at higher frequencies, which is related to the electron transfer process [39]. The semicircle portion diameter can be used to determine the electron transfer resistance (Rct) at the surface of the sensor [40]. The Rct values for bare GCE (538 Ω), GCE/Au (398 Ω), GCE/CNT (278 Ω), and GCE/CNT/Au (171 Ω) electrodes were obtained from the Nyquist plots, by utilizing the equivalent circuit displayed in Figure 2b (inset). Other interesting parameters derived from such analysis may be found in Table S1. The findings indicate that combining CNTs and Au NPs can boost the conductivity performance of the sensor and improve the electron transfer rate of the redox couple on the surface of the electrode. Undoubtedly, these enhancements contribute to improving the analytical properties of the present design, as discussed below.

To acquire information about the underlying mechanisms governing the electrochemical reaction, the GCE/CNT/Au sensor’s peak current response was analyzed at various scan rates ranging from 10 to 400 mV s^−1^. Figure S3a shows the CVs obtained at different scan rates in a ferro/ferricyanide solution, where a clear growth of both peak intensities (cathodic and anodic peaks) is observed at a higher scan rate. Based on the non-linear correlation of both peak currents and the scan rate (see Figure S3b), and the good linear fitting against the square of the scan rate and both peak intensities (see Figure S3c), such findings suggest that the redox reaction is restricted by a diffusion process, where the reactant diffusion rate from the bulk solution to the surface of the electrode is the limiting factor [41].

#### 3.2.1. Evaluation of the Catalytic Activity toward BPA during the Assembling Process

**Figure 3:**
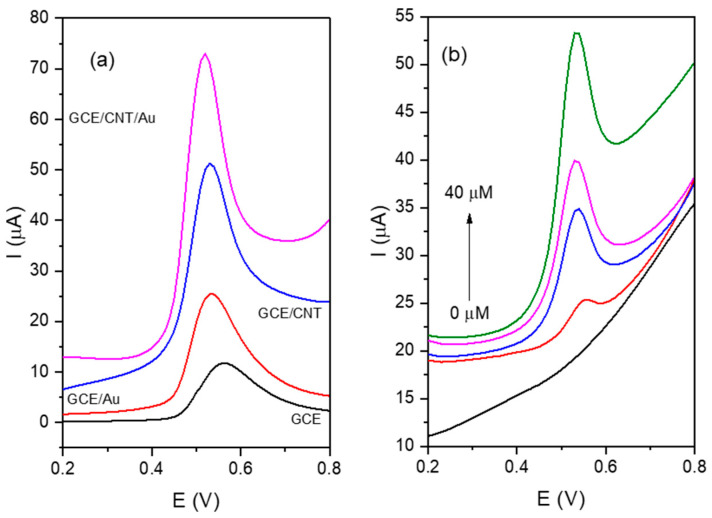
(**a**) Raw differential pulse voltammograms for different electrode configurations in 0.01 M PBS (pH 7.5) containing 0.1 mM BPA. (**b**) Raw differential pulse voltammograms for GCE/CNT/Au electrodes at different BPA concentrations (from the bottom to top: 0, 2, 10, 20, and 40 µM BPA).

The sensor response of the GCE/CNT/Au electrode to BPA was evaluated and compared with the simplest configuration. Figure 3a illustrates the DPV curves for the different electrode configurations against 100 µM BPA, where a clear peak increase is observed during the nanostructuration process, being the sequence of sensitivities of the electrode GCE<GCE/Au<GCE/CNT<GCE/CNT/Au. In addition, a clear shift to lower potential is observed when such nanomaterials are included in the sensor design. Both results confirm the beneficial effect of the use of electrocatalytic materials such as Au NPs and CNTs for enhancing the electrode sensitivity for the determination of BPA. The highest sensitivity obtained for the proposed configuration (GCE/CNT/Au) could be attributed to both the influence of the adsorption accumulation of BPA on the surface of the electrode and the synergetic catalytic effect of combining CNT and Au NPs. In addition, the DPV curves for the GCE/CNT/Au electrode (Figure 3b) exhibited a gradual increase in anodic peak current as the concentration of BPA increases (for details, see below).

#### 3.2.2. Effect of the Scan Rate

The electrochemical mechanism for BPA detection was investigated by obtaining the CV sensor response against 50 μM BPA at different scan rates ranging from 10 to 300 mV (Figure S4a). The anodic current peak (*I_pa_*) rises with the increase in the scan rate (Figure S4b). The linear equation for this relationship can be expressed as *I_pa_* = 0.0383 *v* + 2.946 (R^2^ = 0.993). On the other hand, the peak potential (*E_pa_*) was found to shift positively with the increase in scan rate (Figure S4c). The relationship between *E_pa_* and scan rate can be expressed as *E_pa_* = 0.0231 *ln v* + 0.608 (R^2^ = 0.997). Both results suggest that the BPA oxidation reaction on the surface of the electrode was a process limited by adsorption (surface-controlled electrode process) [42,43]. Assuming an irreversible redox process and using the Laviron equation (Equation (1)) [44]:

(1) $$E_{pa}=E^{o}+(\frac{RT}{\alpha nF})ln\left(\frac{RTk^{0}}{\alpha nF}\right)+\left(\frac{RT}{\alpha nF}\right)ln\;v=cte+\left(\frac{RT}{\alpha nF}\right)ln\;v$$

where n is the number of transfer electrons, *R*, *T*, and *F* are the molar gas constant, Kelvin temperature, and Faraday constant, respectively, and *α* is the electron transfer coefficient (assumed to be 0.5). By employing this equation, the transferred electron number during the reaction was derived from the slope of Figure S4c, being its value ca. 2 [45]. Thus, it was determined that BPA electrooxidation on GCE/CNT/Au electrode was a two-electron process, in good agreement with Scheme 1.

#### 3.2.3. Effect of the pH on the Determination of BPA

**Figure 4:**
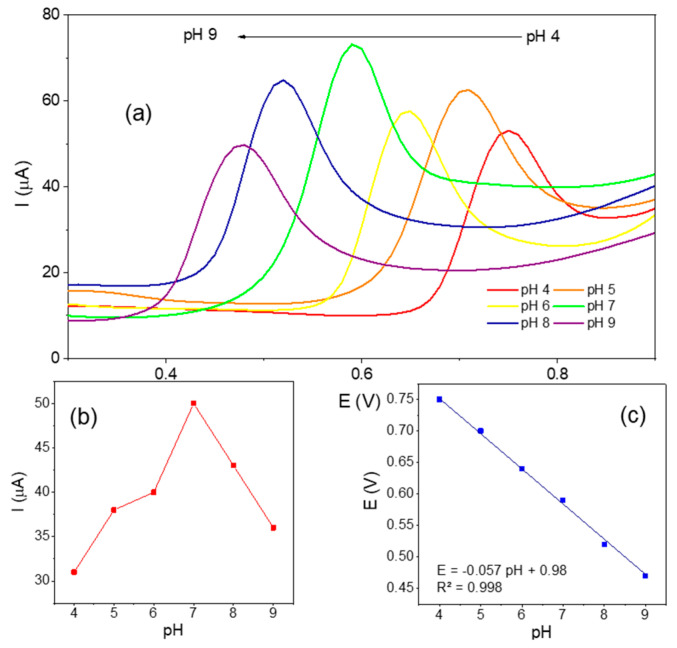
(**a**) Differential pulse voltammograms (DPV) obtained for GCE/CNT/Au electrode against 0.1 mM BPA at different pH values. (**b**) Peak current values obtained for previous data. (**c**) Linear relationship of the potential (E(v)) of the previous DPV data against pH.

The pH influence on the electrochemical sensor was studied using DPV. To this end, the sensor response was obtained in the pH range of 4.0–9.0 (Figure 4a). The highest peak current of BPA was observed at pH 7.0 (Figure 4b), which was chosen to maximize the electrode sensitivity and used for further analysis [46]. On the other hand, the oxidation potential shifted linearly towards negative potentials when the pH values were increased, and such phenomena suggest that protons are implicated in the electrochemical oxidation reaction (Figure 4c) [47]. The linear relationship obtained between pH and the potential value of the peak current and equal to E(V) = −0.057 pH + 0.98 (R^2^ = 0.998), suggests that the number of electrons (see Scheme 1) was equivalent to the number of protons involved (2e^−^2H^+^ process) in the electrode reaction [42,48].

#### 3.2.4. Analytical Performance for GCE/CNT/Au

**Figure 5:**
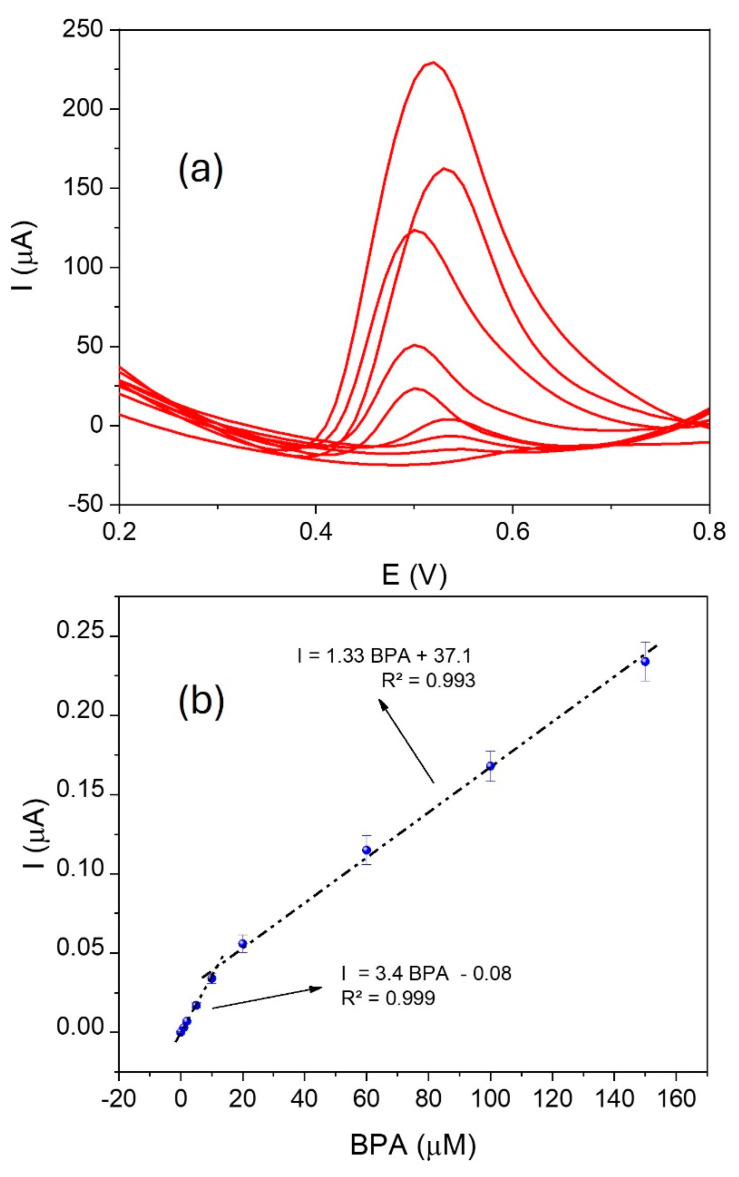
(**a**) Differential pulse voltammograms (DPV) for GCE/CNT/Au electrodes (after background subtraction) at different BPA concentrations in the range 0–150 µM in 0.01 M PBS (pH 7.5). (**b**) Calibration curve for BPA, in the two linear regions obtained, error bars represent the standard deviation (SD, n = 3).

The analytical evaluation of the sensor was conducted using DPV. The voltage range of DPV was tested from +0.2 to +0.8 V with an amplitude of 50 mV, scan rate 0.025 V/s, and pulse width of 0.02 s in BPA solution. For this purpose, various samples containing BPA in the concentration range of 0–150 µM were prepared. Figure 5a displays the current trace, obtained from +0.2 to +0.8 V, after background subtraction. As the BPA concentration grew, the peak current also increased, indicating a positive correlation. At a low BPA concentration (see Figure 5b), a good linear regression was obtained: I (µA) = 3.4 BPA (µM) −0.08 (R^2^ = 0.999), with a detection limit (LOD; S/N = 3) of about 0.31 µM. Such value is in agreement with previous works under similar conditions [49,50,51]. However, the electropolymerization effect due to the by-product reaction with the sensor surface significantly changed the sensor properties at approximately 10 μM, and similar results have been previously described. For details about mechanism detection and electropolymerization effects, see Kim et al. [51]. Therefore, a second linear region (see Figure 5b) can be expressed by the next equation: I (µA) = 1.33 BPA(µM) + 37.1 (R^2^ = 0.993) for the range of concentration from 10 to 150 μM.

To enhance the sensitivity of the method, the sensor stability, and its useful life, several experiments were carried out. Initially, an attempt was made to improve sensitivity and decrease the LOD through a pre-concentration step. In this approach the sensors were exposed to different BPA concentrations for 30 m, followed by the DPV step in PBS. However, no significant changes in the LOD were observed. To enhance the sensor stability, the electropolymerization reaction was studied using CV. It was observed that at very low BPA concentrations, the CV profile remained constant during several cycles without deterioration. However, at higher concentrations, the current decreased due to the electrode fouling. Figure S5 shows the CVs obtained from a continuous scan at 50 μM BPA. Following the first cycle, the current peak decreased ca. 80%. The generation of secondary oxidation products fouls the sensor surface, creating a typical problem that needs to be solved for reliable applications [52]. To address this issue, various approaches were explored such as incorporating polymeric films like Nafion and PEI or washing the electrode with organic solvent. Unfortunately, the sensor sensitivity was not recovered.

### 3.3. Characterization and Analytical Properties of SPE/CNT/Au Electrodes: For FIA Applications

Due to the main problems encountered with the GCEs (reusability, high LOD, and surface deterioration), an alternative approach was implemented. In this regard, SPEs were appropriately modified with 10 µL of CNT (1 mg/mL) and Au NPs (deposited under the optimized condition described above). After that, the SPE/CNT/Au electrode was introduced in a flow cell. Under flow conditions, the BPA sample solution flows over the electrode surface at high speeds for a few seconds. Afterward, the carrier solution washes the electrode surface. Operating at high flow conditions may reduce the diffusion layer, improving the analytical signal and accelerating the response time of the sensor. Additionally, the continuous flow of both the sample and the carrier solutions helps to prevent electrode deterioration caused by by-products through the washing effect.

#### 3.3.1. Analytical Performance for SPE/CNT/Au under FIA Approach

Under the FIA approach, constant potential amperometry (CPA) was chosen to follow the electrochemical oxidation of BPA. The main analytical parameters were studied and optimized (not shown), resulting in the following optimized values: applied potential (+0.6 V), flow rate (1.5 mL/min), and pH of the solution (7).

**Figure 6:**
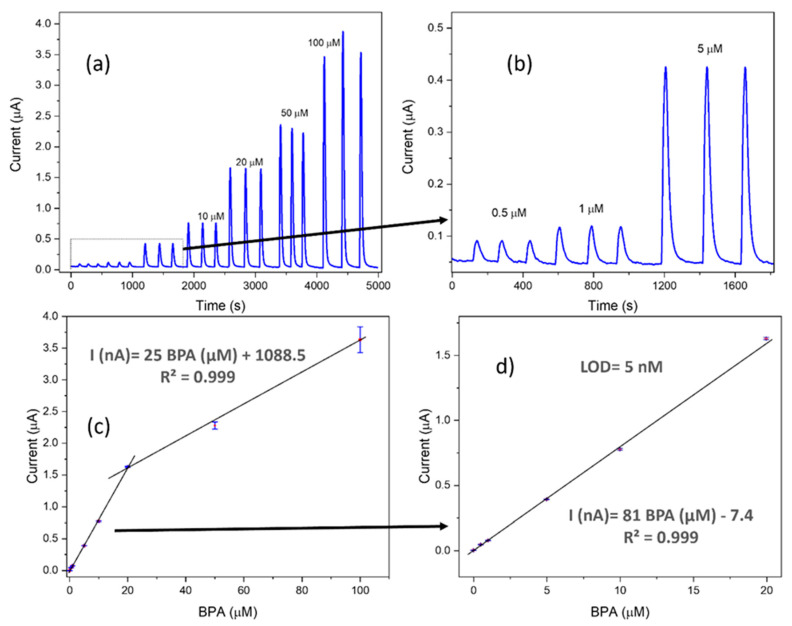
(**a**,**b**) Raw amperometric measurements using the FIA system, with an applied potential of 0.6 V and triplicate BPA injections. Both injected samples and carrier solutions (PBS solution with 0.1 M^−1^) were adjusted to a pH of 7. (**c**,**d**) Calibration curve for the amperometric peak currents against BPA concentration, error bars represent the SD (n = 3).

In Figure 6a, the current trace obtained at +0.5 V vs. the Ag pseudo-reference electrode is presented. The current registered by the sensor increased after each injection and with the increase in BPA concentration, indicating a positive correlation. At low BPA concentrations, a linear regression of I (nA) = 81 BPA (µM)–7.4 (R^2^ = 0.999) was obtained, with an LOD of approximately 5 nM (see Figure 6b). The last value is lower than the previous LOD reported by using the DPV method and confirmed the superior analytical properties of the flow method. As previously described, the sensor sensitivity decreased at higher concentrations; however, we extend the first linear range up to 20 µM. Thus, the second linear region can be defined by the following equation: I (nA) = 25 BPA(µM) + 1088.5 (R^2^ = 0.999) for the concentration range of 20 to 150 μM (see Figure 6b). As shown in Figure 6a, the current peaks for each BPA concentration are quite reproducible, so we ascribed the lower sensitivity of the sensor at higher concentrations because of the saturation of the active center on the sensor surface. This affirmation was confirmed by observing that after each calibration curve, the SPE/CNT/Au electrode was successfully employed in other experiments without any loss of sensitivity. This result contrasts with the 80% signal loss obtained with the DPV method (see above) and confirms our earlier hypothesis, where the continuous flow and ongoing washing of the electrode surface prevent sensor degradation.

According to Table 1, the analytical capabilities of the GCE/CNT/Au and SPE/CNT/Au sensors were compared. Our results revealed that both sensors demonstrated remarkable sensitivity, an extensive linear range, and a low LOD towards BPA. However, the analytical performance of the SPE/CNT/Au in combination with FIA devices yielded satisfactory results, with lower LODs than those reported for other electrochemical sensors (see Table 1). While some electrocatalytic parameters are lower compared to previously reported electrodes, it is important to emphasize the satisfactory outcomes achieved with our detection system. The combination of screen-printed electrodes, offering a robust blend of efficiency and functional versatility, with a flow injection system (FIA) for efficient process automation, enhances both productivity and reproducibility of analysis [53]. Additionally, the FIA system's low reagent consumption and reduced waste generation contribute to its environmental friendliness and long-term economic viability [54]. Despite the initial appearance of less promising electrocatalytic results, the fundamental innovation lies in the electrode design, which allows for easy adaptation and functionalization, offering potential applications to improve detection selectivity and sensitivity. The last result confirmed the utility of combining both strategies, the electrochemical readout and FIA approach, for analytical applications.

Previous research consistently utilizes CNTs to enhance the sensitivity and selectivity of electrochemical sensors for detecting BPA, highlighting their effectiveness in developing analytical methods for identifying environmental contaminants. For instance, Tu et al. [64] employed an electrochemical method using a glassy carbon electrode modified with a hybrid film of multi-walled carbon nanotubes and gold NPs. Similarly, Messaoud et al. [63] also utilized multi-walled carbon nanotubes modified with gold nanoparticles for BPA detection. While Tu et al. present an LOD of 7.5 nM and a narrow linear range, and Messaoud et al. exhibit a slightly lower LOD of 4 nM but a linear range covering unusual concentrations, our work achieves a competitive LOD of 5 nM and a wide linear range of up to 20 μM. This suggests that our methodology offers an optimal balance between sensitivity and the ability to detect both low and high concentrations of BPA, positioning it as a versatile and effective option for BPA detection applications.

When comparing these previous approaches to the present work, distinct advantages emerge that make our methodology superior. Particularly noteworthy is the adoption of screen-printed electrodes instead of glassy carbon electrodes, as reported in our study. This modification effectively mitigates electrode degradation issues, thereby enhancing system stability and reproducibility. Another notable enhancement is the incorporation of a flow injection device, enabling automation and routine analysis, which accelerates sample processing without compromising electrode integrity. In summary, these refinements establish our proposed method as a more advanced and efficient option compared to previously introduced approaches. The utilization of SPEs to enhance system stability and reproducibility, coupled with the automation enabled by the flow injection device, positions this methodology as the preferred choice for accurate and sensitive detection of BPA, particularly in critical settings such as water analysis laboratories, where efficiency and reliability are crucial.

#### 3.3.2. Interference, Stability, and Reproducibility Tests

The selectivity of the SPE/CNT/Au sensor for BPA was investigated considering the presence of potentially interfering substances (see Table 2). The concentrations of these potential interferences were 100-fold higher than BPA to guarantee the selectivity towards this analyte. The substances examined included glucose, fructose, lactose, sucrose, urea, ethanol, methanol, phenol, 2-aminophenol, 4-aminophenol, KNO_3_, MgSO_4_, NH_4_Cl, and Na_2_CO_3_. As expected for most of the organic compounds and inorganic salts, the sensor showed adequate tolerance (<10%) for the determination of BPA at 100-fold concentration. Only methanol and sucrose showed slightly higher values. On the other hand, easily oxidized compounds such as phenol, 2-aminophenol, and 4-aminophenol caused high interference. However, the presence of phenol and its derivatives in polycarbonate-containing plastics, for agro-food applications, is not expected.

The stability, repeatability, and reproducibility of the modified sensors were also studied under FIA applications. The response for five injections (1 μM BPA) presented a relative standard deviation (RSD) value of about 1.8%, which confirms the excellent sensing properties. In addition, the reproducibility (using different sensors assembled in the same way, n = 4) presented an RSD value of about 4.3%, ratifying the suitable construction of the sensor (adequate electrodeposition method and good homogeneity of the nanocomposite). The stability of the sensor during the FIA measurements was evaluated for almost 1 h with 18 continuous injections (1 μM BPA). The current peak repeatability presented an RSD value of ca. 3.3% (see Figure S6) and remained at 99% of the initial response at the end of the test.

#### 3.3.3. Analysis Applications

The practical applications and reliability of using the SPE/CNT/Au electrodes in combination with FIA were evaluated in real samples. To do this, three different water samples were analyzed: bottled water in PET (sample 1), polycarbonate (sample 2) containers, and tap water (sample 3) obtained in our laboratory. Firstly, to minimize the possible matrix effects, the standard addition method and recovery studies were used (see Figure S7). Moreover, water samples and standard solutions were properly buffered with sodium phosphate buffer salts at pH 7. Standard addition graphs displayed an excellent linear relationship between the peak current and different spiked BPA concentrations (see Table 3). The BPA contents in the water samples were calculated from the x-intercept of the standard addition graphs. The recoveries of the BPA standard introduced to the water samples ranged from 95 to 105%. All these results confirmed the reliability of the proposed BPA assay for water samples. Finally, the same analyses were conducted using a conventional calibration method and the current peak of the non-spiked and spiked water samples were introduced in the experimental calibration curve to interpolate the BPA concentration. Similar results were obtained and confirmed that, with these types of samples, the matrix effect is negligible. Therefore, a more efficient and time-efficient assay may be employed for water analysis. 

## 4. Conclusions

In summary, the application of gold nanoparticles modified with multi-walled carbon nanotubes (CNT/AuNPs) has been successfully employed in the determination of BPA, yielding satisfactory results. The electrochemical response of the sensors was investigated using differential pulse voltammetry (DPV) for GCEs and constant potential amperometry (CPA) for SPEs. Optimization of the main analytical parameters was carried out. Addressing challenges such as sensor degradation and high LOD was achieved by incorporating the modified SPEs into a flow injection device, resulting in a low LOD of 5 nM and a linear range of up to 20 μM. These improvements can be attributed to better BPA access to the sensor surface, a higher signal/noise ratio, improved reproducibility, and continuous washing of the sensor surface by the carrier buffer. The selectivity of the SPE/CNT/Au sensor towards BPA was studied, demonstrating adequate tolerance for potential interfering substances. Stability, repeatability, and reproducibility of the modified sensors were thoroughly investigated under flow injection analysis (FIA) conditions. The sensors maintained 99% of the initial response after 18 continuous injections during the 1-hour stability test. Practical application and reliability of the SPE/CNT/Au electrodes in combination with FIA were demonstrated using real water samples, with recoveries ranging from 95% to 105%. Overall, the results obtained underscore the potential of these platforms for enhancing electrochemical biosensors enabling sensitive, precise, and specific identification of BPA in water samples.

## Figures and Tables

**Table 1:** Comparative analysis of the analytical performance of the proposed sensor for BPA detection with other nanomaterial-modified electrode sensors documented in the literature.

Sensor Material	Detection Method	LoD (nM)	Linear Range (μM)	References
NCNF/CPE ^a^	DPV	50	0.1–60	[55]
GO-MWCNT-βCD/SPE ^b^	LSV	6	0.05–5/5–30	[6]
GDY-cMWCNT/SPE ^c^	LSV	13	0.2–500	[56]
DPNs ^d^	Amp	6.6	0.01–1/1–300	[57]
AuNPs/MoS_2_^e^	CV	5	0.05–100	[58]
Rh_2_O_3_-rGO ^f^	CV	120	0.6–40	[59]
Ni_2_Al-LDH ^g^	DPV	7	0.02–1.51	[8]
GCE/MNPs-rGO-CS ^h^	DPV	170	0.06–11	[1]
GCE/Pt-Gr-CNTs ^i^	DPV	420	0.06–10	[60]
SPCE/PEDOT/NMIMBr ^j^	CV	20	0.1–500	[61]
AuNP/MWCNT/GCE ^k^	DPV	4.3	0.01–0.7	[62]
MWCNT-GNPs/GCE ^l^	DPV	7.5	0.02–20	[63]
MWCNTs@Cu-N-PC ^m^	DPV	5.8	0.01–100	[64]
GCE/CNT/Au	DPV	31	0–10	This work
GCE/CNT/Au	DPV	--	10–160	This work
SPE/CNT/Au	CPA/FIA	5	1–20	This work
SPE/CNT/Au	CPA/FIA	--	20–100	This work

^a.^ NCNF/CPE: nitrogen-doped carbon nanofiber-modified carbon paste electrode. ^b.^ GO-MWCNT-βCD/SPE: graphene oxide and β-cyclodextrin functionalized multi-walled carbon nanotubes. ^c.^ GDY-cMWCNT/SPE: screen-printed electrode modified by graphdiyne and carbon nanotubes. ^d.^ DPNs: dendritic platinum nanoparticles. ^e.^ AuNPs/MoS2: molybdenum disulfide nanoflower–chitosan–Au nanoparticles. ^f.^ Rh2O3-rGO: rhodium oxide and reduced graphene oxide electrode. ^g.^ Ni2Al-LDH: exfoliated Ni2Al layered double hydroxide nanosheet electrode. ^h.^ GCE/MNPs-rGO-CS: glassy carbon electrode modified with magnetic nanoparticles, reduced graphene oxide, and chitosan. ^i.^ GCE/Pt-Gr-CNTs: glassy carbon electrode modified with gold nanoparticles, graphene, and carbon nanotubes. ^j.^ SPCE/PEDOT/NMIMBr: screen-printed carbon electrodes modified with conducting poly(3,4-ethylenedioxythiophene) film (PEDOT) and ionic liquid 1-butyl-3-methylimidazolium bromide (BMIMBr). ^k.^ AuNP/MWCNT/GCE: gold nanoparticles and multi-walled carbon nanotubes composite modified glassy carbon electrode. ^l.^ MWCNT-GNPs/GCE: glassy carbon electrode modified with multi-walled carbon nanotubes–gold nanoparticles hybrid film. ^m.^ MWCNTs@Cu-N-PC: nanohybrid nitrogen coordinated copper co-doped multi-walled carbon nanotubes.

**Table 2:** Interference effects on the determination of 5 μM BPA in phosphate buffer 0.1 M pH 7.0.

Interference	Concentration (mM)	Signal Change (%)	RSD ^a^ (%)
Glucose	0.5	−3.8	1.10
Fructose	0.5	−1.8	2.74
Lactose	0.5	1.1	1.57
Sucrose	0.5	−11.2	2.15
Urea	0.5	−8.1	0.50
Ethanol	0.5	2.3	0.93
Methanol	0.5	−13.0	0.41
KNO_3_	0.5	−1.6	1.64
MgSO_4_	0.5	−8.8	0.39
NH_4_Cl	0.5	−2.0	0.95
Na_2_CO_3_	0.5	−2.4	1.30

^a.^ Relative standard deviation (RSD) for three replicates.

**Table 3:** Standard addition results of the recovery tests obtained by the proposed electrochemical method.

Sample ^a^	Spiked (μM)	Total ^b^ (μM)	Recovery ^c^ (%)	RSD ^d^ (%)
1	0	2.14	-	1.5
	2	4.20	101.4	1.5
	2.5	4.47	96.3	4.6
	5	6.94	97.2	0.45
	6	7.73	95.0	2.4
2	0	3.09	-	4.2
	2	5.16	101.4	2.7
	2.5	5.89	105.4	4.1
	5	7.98	98.6	3.6
	6	8.87	97.6	2.1
3	0	4.39	-	4.6
	2	6.10	95.5	3.4
	2.5	6.57	95.4	1.9
	5	9.49	101.1	0.95
	6	10.3	99.1	2.3

^a.^ 1: polyethylene terephthalate drink bottle; 2: polycarbonate water bottle, 3: tap water. ^b.^ The total measured concentration after addition of known amount of standard in the real sample. ^c.^ Recovery (%) = [measured concentration in real sample)/(spiked + BPA background concentration)] × 100. ^d.^ Three replicates were performed for each determination.

## Data Availability

Data are contained within the article.

## Supplementary Materials

The following supporting information can be downloaded at: https://www.mdpi.com/article/10.3390/s24082570/s1, Figure S1: Typical potential transient obtained during galvanostatic Au deposition on GCE; Figure S2: X-ray diffractogram for a CNT/Au nanocomposite deposited onto an indium-tin oxide (ITO) electrode; Figure S3: Cyclic voltammograms of the GCE/CNT/Au electrode (a) at different scan rate in 5 mM ferro/ferricyanide solution implemented with 0.1 M KCl; Figure S4: (a) Cyclic voltammograms of the GCE/CNT/Au electrode at different scan rate (*v*): 10, 30, 80, 120, 200, 300 mV/s, in 50 µM BPA 0.01 M PBS (pH = 7); Figure S5: Cyclic voltammograms (CV) of GCE/CNT/Au for consecutive scans in a 50 µM BPA solution in 0.01 M phosphate buffer solution; Figure S6: Eighteen successive injection of 1 µM BPA solution in 0.01 M phosphate buffer solution); Figure S7: Standard calibration curve and water samples concentration estimation (sample 1) from the x-intercept; Table S1: Parameters values obtained from the EIS data fitting for different sensor configurations.

## References

[1] Zhang Y., Cheng Y., Zhou Y., Li B., Gu W., Shi X., Xian Y. (2013). Electrochemical sensor for bisphenol A based on magnetic nanoparticles decorated reduced graphene oxide. Talanta.

[2] Niu X., Yang W., Wang G., Ren J., Guo H., Gao J. (2013). A novel electrochemical sensor of bisphenol A based on stacked graphene nanofibers/gold nanoparticles composite modified glassy carbon electrode. Electrochim. Acta.

[3] Xu W., Zhang Y., Yin X., Zhang L., Cao Y., Ni X., Huang W. (2021). Highly sensitive electrochemical BPA sensor based on titanium nitride-reduced graphene oxide composite and core-shell molecular imprinting particles. Anal. Bioanal. Chem..

[4] Ginter-Kramarczyk D., Zembrzuska J., Kruszelnicka I., Zając-Woźnialis A., Ciślak M. (2022). Influence of Temperature on the Quantity of Bisphenol A in Bottled Drinking Water. Int. J. Environ. Res. Public Health.

[5] da Silva Costa R., Sainara Maia Fernandes T., de Sousa Almeida E., Tomé Oliveira J., Carvalho Guedes J.A., Julião Zocolo G., Wagner de Sousa F., do Nascimento R.F. (2021). Potential risk of BPA and phthalates in commercial water bottles: A minireview. J. Water Health.

[6] Alam A.U., Deen M.J. (2020). Bisphenol A Electrochemical Sensor Using Graphene Oxide and β-Cyclodextrin-Functionalized Multi-Walled Carbon Nanotubes. Anal. Chem..

[7] Tarafdar A., Sirohi R., Balakumaran P.A., Reshmy R., Madhavan A., Sindhu R., Binod P., Kumar Y., Kumar D., Sim S.J. (2022). The hazardous threat of Bisphenol A: Toxicity, detection and remediation. J. Hazard. Mater..

[8] Zhan T., Song Y., Tan Z., Hou W. (2017). Electrochemical bisphenol A sensor based on exfoliated Ni2Al-layered double hydroxide nanosheets modified electrode. Sens. Actuators B Chem..

[9] Zhang J., Xu X., Chen L. (2018). An ultrasensitive electrochemical bisphenol A sensor based on hierarchical Ce-metal-organic framework modified with cetyltrimethylammonium bromide. Sens. Actuators B Chem..

[10] Ma Y., Guo J., Chen Y., Yi Y., Zhu G. (2021). Electrochemical sensing of phenolics based on copper/cobalt/nitrogen co-doped hollow nanocarbon spheres. J. Electroanal. Chem..

[11] Manfo F.P., Jubendradass R., Nantia E.A., Moundipa P.F., Mathur P.P. (2014). Adverse effects of bisphenol A on male reproductive function. Rev. Environ. Contam. Toxicol..

[12] Rochester J.R. (2013). Bisphenol A and human health: A review of the literature. Reprod. Toxicol..

[13] Ohore O.E., Zhang S. (2019). Endocrine disrupting effects of bisphenol A exposure and recent advances on its removal by water treatment systems. A review. Sci. Afr..

[14] (2011). Regulation (EU), No.10/2011 of 14 January 2011 on Plastic Materials and Articles Intended to Come into Contact with Food. https://eur-lex.europa.eu/eli/reg/2011/10/oj.

[15] Ma W., Wan S., Lin C., Lin X., Xie Z. (2020). Towards online specific recognition and sensitive analysis of bisphenol A by using AuNPs@aptamer hybrid-silica affinity monolithic column with LC-MS. Talanta.

[16] Martín-Pozo L., Martín-Bueno J., Moscoso-Ruiz I., Zafra-Gómez A., Sarma H., Dominguez D.C., Lee W.-Y. (2022). Chapter 18—Methods of bisphenol A detection by gas chromatography and mass spectrometry (GC-Ms) in human breast milk and foodstuff. Emerging Contaminants in the Environment.

[17] Owczarek K., Waraksa E., Kłodzińska E., Zrobok Y., Ozimek M., Rachoń D., Kudłak B., Wasik A., Mazerska Z. (2022). Validated GC–MS method for determination of bisphenol a and its five analogues in dietary and nutritional supplements. Microchem. J..

[18] Vidal R.B.P., Ibañez G.A., Escandar G.M. (2015). Chemometrics-assisted cyclodextrin-enhanced excitation–emission fluorescence spectroscopy for the simultaneous green determination of bisphenol A and nonylphenol in plastics. Talanta.

[19] Hou C., Zhao L., Geng F., Wang D., Guo L.-H. (2016). Donor/acceptor nanoparticle pair-based singlet oxygen channeling homogenous chemiluminescence immunoassay for quantitative determination of bisphenol A. Anal. Bioanal. Chem..

[20] Lei Y., Fang L., Hamid Akash M.S., Liu Z., Shi W., Chen S. (2013). Development and comparison of two competitive ELISAs for the detection of bisphenol A in human urine. Anal. Methods.

[21] Zhang C., Yi Y., Zhou Y., Zhou R., Zhu G. (2024). Homogeneous electroanalysis coupled with colorimetry dual-mode sensing of silver ion in water based on target-inhibited peroxidase activity of Pt@ZIF-8 nanozyme. Microchem. J..

[22] Liu X., Yao Y., Ying Y., Ping J. (2019). Recent advances in nanomaterial-enabled screen-printed electrochemical sensors for heavy metal detection. TrAC Trends Anal. Chem..

[23] Dhaffouli A., Salazar-Carballo P.A., Carinelli S., Holzinger M., Barhoumi H. (2024). Improved electrochemical sensor using functionalized silica nanoparticles (SiO2-APTES) for high selectivity detection of lead ions. Mater. Chem. Phys..

[24] Baluta S., Meloni F., Halicka K., Szyszka A., Zucca A., Pilo M.I., Cabaj J. (2022). Differential pulse voltammetry and chronoamperometry as analytical tools for epinephrine detection using a tyrosinase-based electrochemical biosensor. RSC Adv..

[25] Jebril S., Cubillana-Aguilera L., Palacios-Santander J.M., Dridi C. (2021). A novel electrochemical sensor modified with green gold sononanoparticles and carbon black nanocomposite for bisphenol A detection. Mater. Sci. Eng. B.

[26] Zhang J., Xu X., Chen Z. (2018). A highly sensitive electrochemical sensor for bisphenol A using cetyltrimethylammonium bromide functionalized carbon nanohorn modified electrode. Ionics.

[27] Zhang Y., Lei Y., Lu H., Shi L., Wang P., Ali Z., Li J. (2021). Electrochemical detection of bisphenols in food: A review. Food Chem..

[28] Aralekallu S., Palanna M., Hadimani S., Prabhu C.P.K., Sajjan V.A., Thotiyl M.O., Sannegowda L.K. (2020). Biologically inspired catalyst for electrochemical reduction of hazardous hexavalent chromium. Dalton Trans..

[29] Sajjan V.A., Aralekallu S., Nemakal M., Palanna M., Keshavananda Prabhu C.P., Koodlur Sannegowda L. (2021). Nanomolar detection of 4-nitrophenol using Schiff-base phthalocyanine. Microchem. J..

[30] Bounegru A.V., Apetrei C. (2020). Carbonaceous Nanomaterials Employed in the Development of Electrochemical Sensors Based on Screen-Printing Technique—A Review. Catalysts.

[31] Musa A.M., Kiely J., Luxton R., Honeychurch K.C. (2023). An Electrochemical Screen-Printed Sensor Based on Gold-Nanoparticle-Decorated Reduced Graphene Oxide-Carbon Nanotubes Composites for the Determination of 17-beta Estradiol. Biosensors.

[32] Fritea L., Banica F., Costea T.O., Moldovan L., Dobjanschi L., Muresan M., Cavalu S. (2021). Metal Nanoparticles and Carbon-Based Nanomaterials for Improved Performances of Electrochemical (Bio)Sensors with Biomedical Applications. Materials.

[33] Otero F., Magner E. (2020). Biosensors—Recent Advances and Future Challenges in Electrode Materials. Sensors.

[34] de Faria L.V., Lisboa T.P., de Farias D.M., Araujo F.M., Machado M.M., de Sousa R.A., Matos M.A.C., Munoz R.A.A., Matos R.C. (2020). Direct analysis of ascorbic acid in food beverage samples by flow injection analysis using reduced graphene oxide sensor. Food Chem..

[35] Trojanowicz M., Pyszynska M. (2022). Flow-Injection Methods in Water Analysis-Recent Developments. Molecules.

[36] Martínez-Paredes G., González-García M.B., Costa-García A. (2009). In situ electrochemical generation of gold nanostructured screen-printed carbon electrodes. Application to the detection of lead underpotential deposition. Electrochim. Acta.

[37] Pérez-Fernández B., Martín-Yerga D., Costa-García A. (2017). Galvanostatic electrodeposition of copper nanoparticles on screen-printed carbon electrodes and their application for reducing sugars determination. Talanta.

[38] Rivera-Gavidia L.M., Fernández de la Puente I., Hernández-Rodríguez M.A., Celorrio V., Sebastián D., Lázaro M.J., Pastor E., García G. (2020). Bi-functional carbon-based catalysts for unitized regenerative fuel cells. J. Catal..

[39] Carinelli S., Fernández I., Luis González-Mora J., Salazar-Carballo P.A. (2022). Hemoglobin-modified nanoparticles for electrochemical determination of haptoglobin: Application in bovine mastitis diagnosis. Microchem. J..

[40] Fernández I., González-Mora J.L., Lorenzo-Luis P., Villalonga R., Salazar-Carballo P.A. (2020). Nickel oxide nanoparticles-modified glassy carbon electrodes for non-enzymatic determination of total sugars in commercial beverages. Microchem. J..

[41] Zhang S., Zhao W., Liu C., Zeng J., He Z., Wang C., Yuan W., Wang Q. (2024). Flower-like CoO nanowire-decorated Ni foam: A non-invasive electrochemical biosensor for glucose detection in human saliva. Appl. Mater. Today.

[42] Baghayeri M., Nazarzadeh Zare E., Mansour Lakouraj M. (2014). A simple hydrogen peroxide biosensor based on a novel electro-magnetic poly(p-phenylenediamine)@Fe3O4 nanocomposite. Biosens. Bioelectron..

[43] Xing Y., Zhou S., Wu G., Wang C., Yuan X., Feng Q., Zhu X., Qu J. (2021). A sensitive electrochemical sensor for bisphenol F detection and its application in evaluating cytotoxicity. Microchem. J..

[44] Laviron E. (1974). Adsorption, autoinhibition and autocatalysis in polarography and in linear potential sweep voltammetry. J. Electroanal. Chem. Interfacial Electrochem..

[45] Ławrywianiec M., Smajdor J., Paczosa-Bator B., Piech R. (2017). High Sensitive Method for Determination of the Toxic Bisphenol A in Food/Beverage Packaging and Thermal Paper Using Glassy Carbon Electrode Modified with Carbon Black Nanoparticles. Food Anal. Methods.

[46] Ben Jaballah M., Ben Messaoud N., Dridi C. (2021). Nanoengineering of new cost-effective nanosensor based on functionalized MWCNT and Ag nanoparticles for sensitive detection of BPA in drinking water. Appl. Phys. A.

[47] Jemmeli D., Dridi C., Abbas M.N., Dempsey E. (2021). Development of highly sensitive and selective bisphenol A sensor based on a cobalt phthalocyanine-modified carbon paste electrode: Application in dairy analysis. Anal. Methods.

[48] Xin X., Sun S., Li H., Wang M., Jia R. (2015). Electrochemical bisphenol A sensor based on core–shell multiwalled carbon nanotubes/graphene oxide nanoribbons. Sens. Actuators B Chem..

[49] Ndlovu T., Arotiba O.A., Sampath S., Krause R.W., Mamba B.B. (2012). An Exfoliated Graphite-Based Bisphenol A Electrochemical Sensor. Sensors.

[50] Li Q., Li H., Du G.-F., Xu Z.-H. (2010). Electrochemical detection of bisphenol A mediated by [Ru(bpy)3]^2+^ on an ITO electrode. J. Hazard. Mater..

[51] Kim M., Song Y.E., Xiong J.-Q., Kim K.-Y., Jang M., Jeon B.-H., Kim J.R. (2021). Electrochemical detection and simultaneous removal of endocrine disruptor, bisphenol A using a carbon felt electrode. J. Electroanal. Chem..

[52] Gugoasa L.A.D. (2020). Review—Electrochemical Sensors for Determination of the Endocrine Disruptor, Bisphenol A. J. Electrochem. Soc..

[53] Trojanowicz M. (2023). Impact of nanotechnology on progress of flow methods in chemical analysis: A review. Anal. Chim. Acta.

[54] Mezaal E.N., Kawther Ahmed S., Tamara Ahmed Abdel K. (2024). A Review using continuous flow injection analysis technique in the determination of several drugs. Ibn AL-Haitham J. Pure Appl. Sci..

[55] Sun J., Liu Y., Lv S., Huang Z., Cui L., Wu T. (2016). An Electrochemical Sensor Based on Nitrogen-doped Carbon Nanofiber for Bisphenol A Determination. Electroanalysis.

[56] He S., Xia H., Chang F. (2022). Enzyme free electrochemical determination of bisphenol A using screen-printed electrode modified by graphdiyne and carbon nanotubes. Microchem. J..

[57] Shim K., Kim J., Shahabuddin M., Yamauchi Y., Hossain M.S.A., Kim J.H. (2018). Efficient wide range electrochemical bisphenol-A sensor by self-supported dendritic platinum nanoparticles on screen-printed carbon electrode. Sens. Actuators B Chem..

[58] Huang K.-J., Liu Y.-J., Liu Y.-M., Wang L.-L. (2014). Molybdenum disulfide nanoflower-chitosan-Au nanoparticles composites based electrochemical sensing platform for bisphenol A determination. J. Hazard. Mater..

[59] Shi R., Yuan X., Liu A., Xu M., Zhao Z. (2018). Determination of Bisphenol A in Beverages by an Electrochemical Sensor Based on Rh2O3/Reduced Graphene Oxide Composites. Appl. Sci..

[60] Zheng Z., Du Y., Wang Z., Feng Q., Wang C. (2013). Pt/graphene–CNTs nanocomposite based electrochemical sensors for the determination of endocrine disruptor bisphenol A in thermal printing papers. Analyst.

[61] Wang J.-Y., Su Y.-L., Wu B.-H., Cheng S.-H. (2016). Reusable electrochemical sensor for bisphenol A based on ionic liquid functionalized conducting polymer platform. Talanta.

[62] Ben Messaoud N., Ghica M.E., Dridi C., Ben Ali M., Brett C.M.A. (2017). Electrochemical sensor based on multiwalled carbon nanotube and gold nanoparticle modified electrode for the sensitive detection of bisphenol A. Sens. Actuators B Chem..

[63] Tu X., Yan L., Luo X., Luo S., Xie Q. (2009). Electroanalysis of Bisphenol A at a Multiwalled Carbon Nanotubes-gold Nanoparticles Modified Glassy Carbon Electrode. Electroanalysis.

[64] Yi Y., Zhang D., An P., Zhu G. (2020). Nitrogen Coordinated Copper Co-Doped Multi-Walled Carbon Nanotubes for High-Efficiency Electrochemical Sensing of Bisphenol A. J. Electrochem. Soc..

