# Supplementary material for: Electrochemical Detection of Bisphenol A Based on Gold Nanoparticles/Multi-Walled Carbon Nanotubes: Applications on Glassy Carbon and Screen Printed Electrodes
Source: Sensors (Basel). 2024 Apr 17;24(8):2570. doi: 10.3390/s24082570 (PMC11054518; doi:10.3390/s24082570)
Supplement: Supplementary file 1 [file sensors-24-02570-s001.zip › sensors-2931194-supplementary.pdf]

Supplementary Materials

Electrochemical Detection of Bisphenol A Based on Gold Nanoparticles/Multi-Walled Carbon Nanotubes: Applications on Glassy Carbon and Screen Printed Electrodes

Maximina Luis-Sunga [1,2], Soledad Carinelli [1,3,*], Gonzalo García [2], José Luis González-Mora [1,3,4] and Pedro A. Salazar-Carballo [1]

1 *Laboratory of Sensors, Biosensors and Advanced Materials, Faculty of Health Sciences, Universidad de La Laguna, Campus de Ofra s/n, 38071 La Laguna, Spain*

2 *Departamento de Química, Instituto Universitario de Materiales y Nanotecnología, Universidad de La Laguna, P.O. Box 456, 38200 La Laguna, Spain*

3 *Departamento de Ciencias Médicas Básicas and Instituto de Tecnologías Biomédicas, Universidad de La Laguna, 38200 La Laguna, Spain*

4 *Instituto Universitario de Neurociencia, Universidad de la Laguna, 38071 Santa Cruz de Tenerife, Spain*

* *Correspondence: scarinel@ull.edu.es*

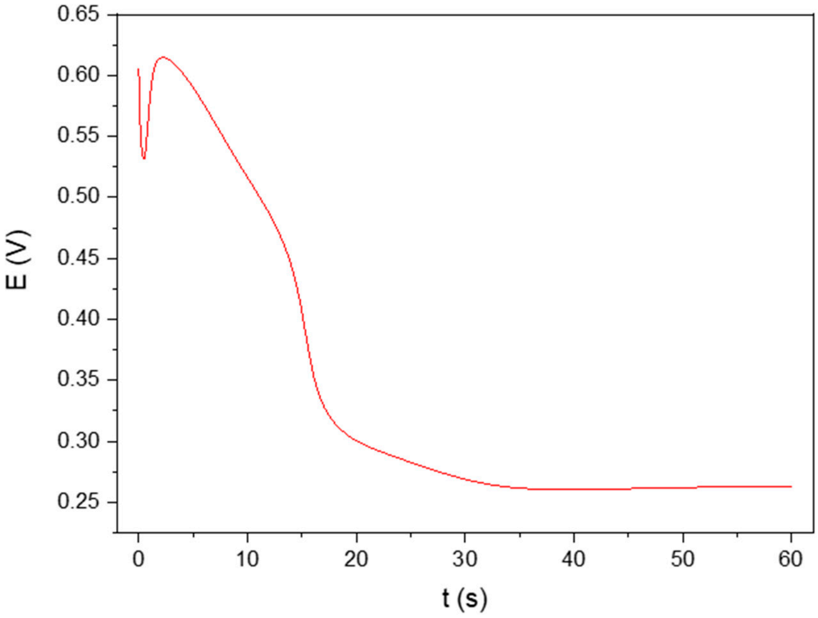

Figure S1. Typical potential transient obtained during galvanostatic Au deposition on GCE.

Platting bath: 3 mM $HAuCl_4$. Applied current pulse: -25 µA for 60 s.

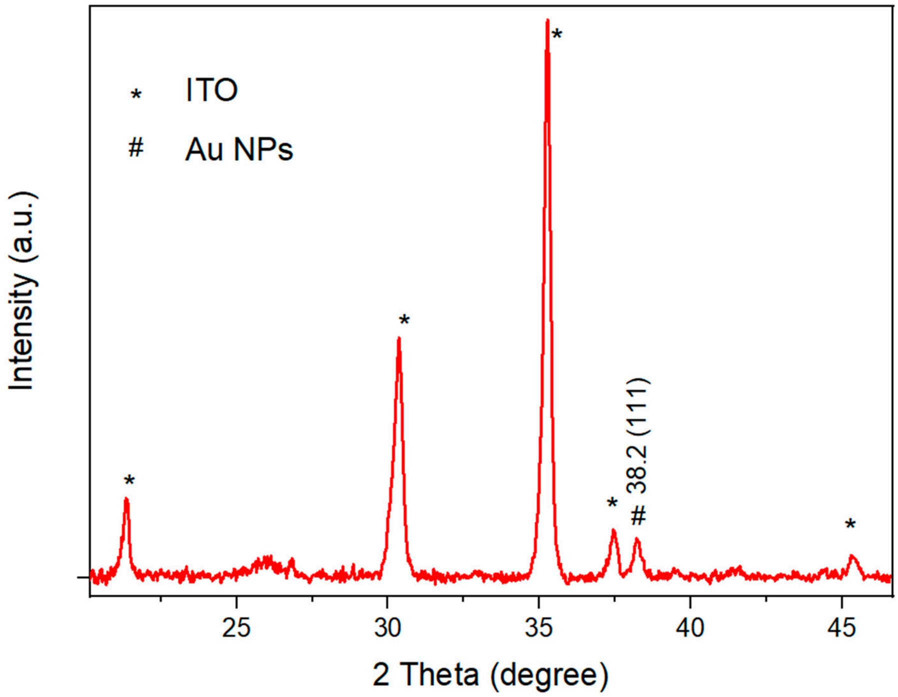

Figure S2. X-ray diffractogram for a CNT/Au nanocomposite deposited onto an indium-tin oxide (ITO) electrode.

Table S1. Parameters values obtained from the EIS data fitting for different sensor configurations.

	GCE	GCE@Au	GCE/CNT	GCE/CNT@Au
**Rs (Ω)**	92.5	89.3	86.8	87.8
**Rct (Ω)**	538	398.4	278.7	171
**W (Kσ)**	0.5	0.5	0.5	0.4
**Q (µT)**	2276	2888	14.4	57.7
**n**	0.9	0.9	0.8	0.7

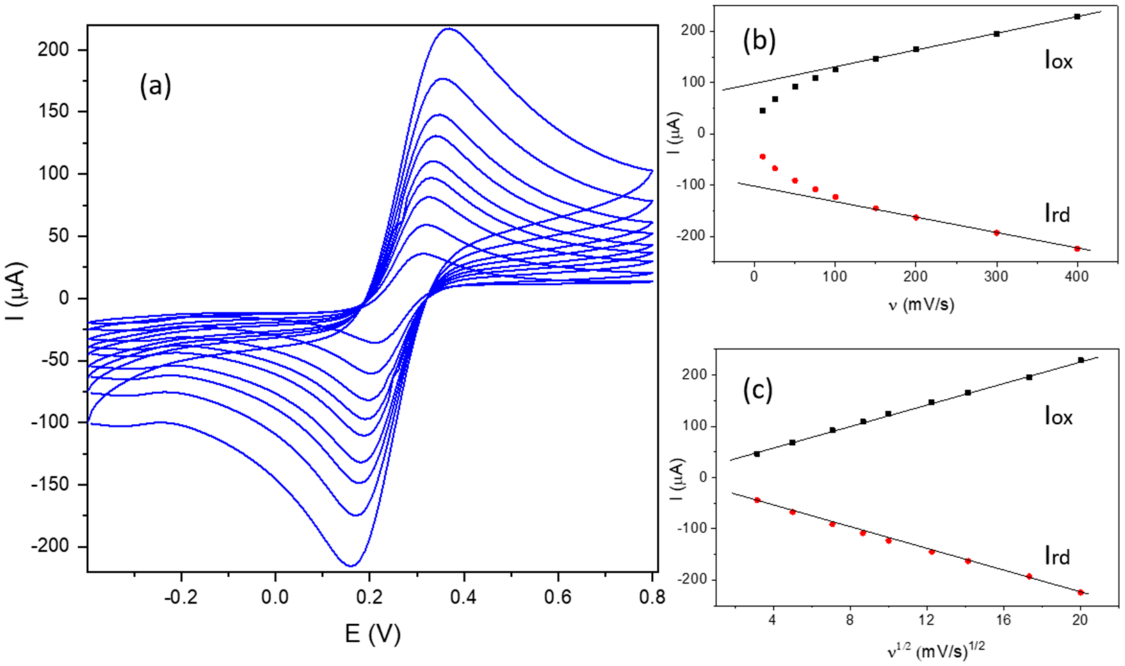

Figure S3. Cyclic voltammograms of the GCE/CNT/Au electrode (a) at different scan rate in 5 mM ferro/ferricyanide solution implemented with 0.1 M KCl. Anodic and cathodic peak intensity currents against de scan rate (b) and the square root of the scan rate (c).

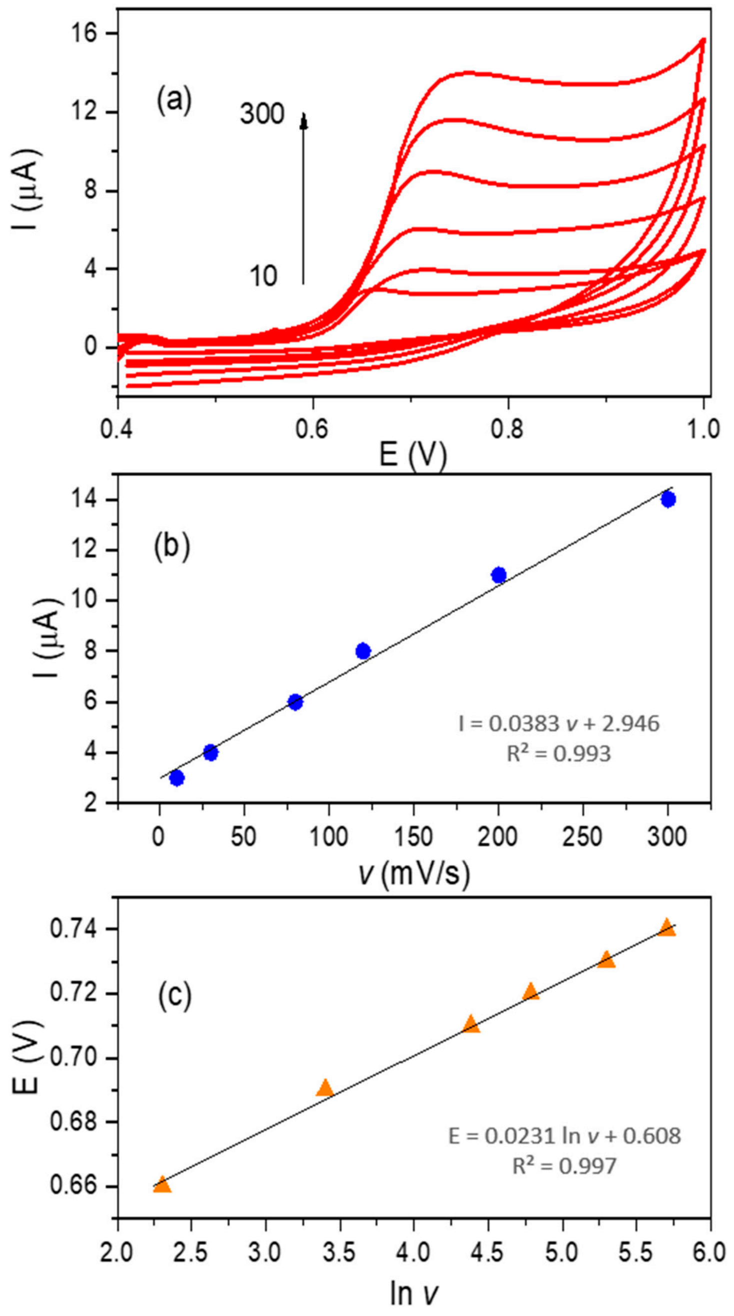

Figure S4. (a) Cyclic voltammograms of the GCE/CNT/Au electrode at different scan rate (*v*): 10, 30, 80, 120, 200, 300 mV/s, in 50 μM BPA 0.01 M PBS (pH = 7). (b) Anodic peak intensity currents against *v.* (c) Anodic peak potential shift against the ln *v*.

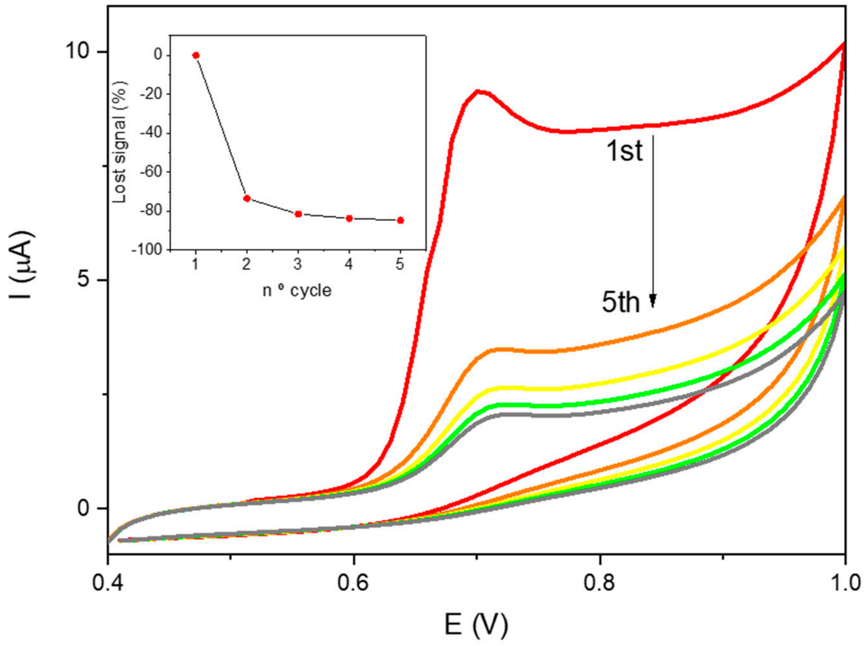

Figure S5. Cyclic voltammograms (CV) of GCE/CNT/Au for consecutive scans in a 50 µM BPA solution in 0.01 M phosphate buffer solution. *Inset: percent of the signal decrease after each cycle.*

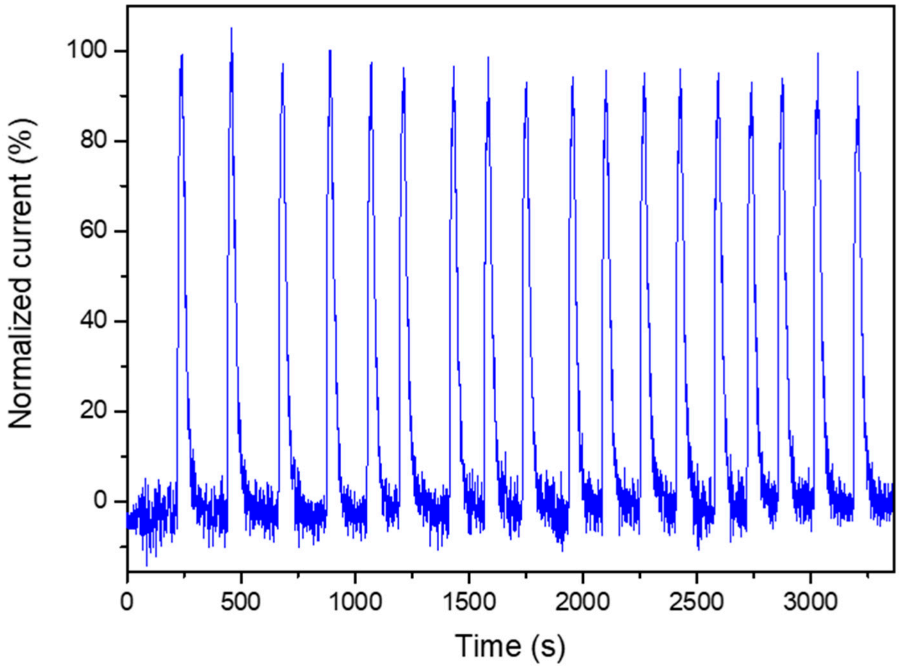

Figure S6. Eighteen successive injection of 1 µM BPA solution in 0.01 M phosphate buffer solution).

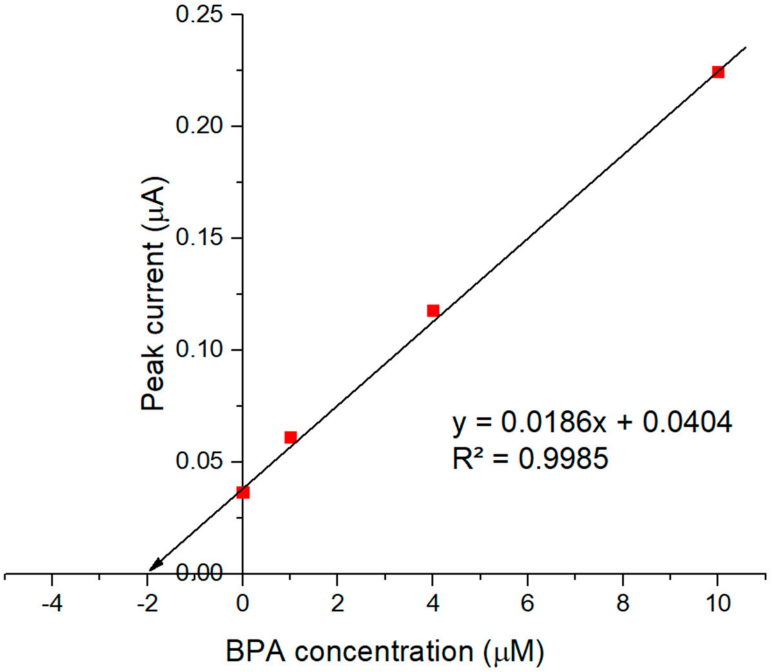

Figure S7. Standard calibration curve and water samples concentration estimation (sample 1) from the x-intercept.
